# The Tobacco Endgame: Is It Possible?

**DOI:** 10.1371/journal.pmed.1001832

**Published:** 2015-05-29

**Authors:** Thomas E. Novotny

**Affiliations:** Division of Epidemiology and Biostatistics, Graduate School of Public Health, San Diego State University, San Diego, California, United States

## Abstract

Reflecting on World No Tobacco Day 2015, Tom Novotny assesses the tobacco control landscape and considers how a tobacco endgame could be a reality.

May 31 is World No Tobacco Day 2015. The day raises an interesting question: is a tobacco endgame possible? The answer, of course, is “it depends.” However, the event provides a reflective opportunity to see just where we are in the extraordinary global battle against the world’s most important preventable health risk. A great deal is in fact happening in pursuit of the endgame, and these efforts are becoming very interesting indeed.

As background, keep in mind these grim statistics: there are more than 1 billion smokers globally, tobacco kills about 6 million of them and their secondhand smoke (SHS)-exposed contacts a year, and, if unchecked, it will kill perhaps a billion people in the 21st century [1]. Could the endgame possibly be near?

In 2012, Professor Ken Warner assembled a group of tobacco control experts in Ann Arbor, Michigan, to discuss this endgame dream and what it would take to make it a reality [2]. The discussion was informed by two précis: that the status quo is unacceptable and that reducing smoking substantially will require something new, bold, and completely different from what is currently in place. In other words, what is being done at present, despite remarkable progress on many fronts, will not be enough to end the epidemic of tobacco-related diseases any time soon.

Now, however, rather than thinking only about “tobacco control,” which implies that humanity should settle for the tobacco epidemic being permanent in some populations or at least at some lower hazard level overall [3], experts are discussing new strategies that are necessary to greatly reduce the global tobacco-related disease burden. These out-of-the box ideas include the following: removing the profit incentive from selling tobacco products by changing the ways in which the market is administered; reducing the level of addictive nicotine to nonaddicting levels in all tobacco products; addressing the supply side of tobacco use by imposing a “sinking lid” on the industry to gradually reduce quotas on sales and production of tobacco products, similar to proposed reductions on greenhouse gas production [4]; establishing truly smoke-free generations by prohibiting possession of tobacco products by all persons born in 2000 or later; and the heretofore unthinkable abolition of tobacco product manufacture and sale.

At the same time, considerable global energy has developed around the world’s first multinational health treaty, implemented under the auspices of the World Health Organization (WHO): the Framework Convention on Tobacco Control (FCTC) [5]. This treaty has been in force for ten years and calls for signatory countries (currently 180) to enact science-based interventions to reduce tobacco use through the six components of the WHO MPOWER tobacco control rubric [6]:

- Monitor tobacco use and prevention policies
- Protect people from tobacco smoke
- Offer help to quit tobacco use
- Warn about the dangers of tobacco
- Enforce bans on tobacco advertising, promotion, and sponsorship
- Raise taxes on tobacco

At the 15th World Conference on Tobacco or Health (WCTOH), held in Abu Dhabi, United Arab Emirates, in March 2015, more than a thousand delegates from around the globe celebrated individual, national, and global progress around MPOWER and the FCTC. Indeed, there was palpable pride in the accomplishments of many nations, but delegates were also reminded of how adaptable the tobacco industry is in working around these successes. Similar to how an infectious disease adapts to antimicrobial therapies with development of drug resistance, the multinational tobacco industry predictably finds new ways to circumvent restrictions and regulations on the use of its products. It is clear to tobacco control advocates that behavioral science is not enough to eliminate tobacco use; today’s tobacco control army needs to include economists, political scientists, toxicologists, trade experts, and environmentalists to fully respond to changing industry tactics. The industry is very good at its job, and thus, the tobacco control community needs to be even better at its job.

One promising yet heavily contested recent effort has been the imposition of plain packaging regulations along with large graphic health warnings on cigarette packages [7]. Bold action using this policy has been taken by Australia, the United Kingdom, Ireland, France, and other countries and has been challenged with newly improvised legal tactics by the tobacco industry [8]. Asserting violations of bilateral trade agreements and intellectual property rights protections under the World Trade Organization treaty obligations and bilateral trade agreements, actions have been brought by the multinational tobacco companies against Australia, which fended off the legal challenge in its Supreme Court, and against much smaller countries such as Uruguay. These small countries would likely incur huge legal costs to defend against a deep-pocketed industry intent on inhibiting them from using proven public health interventions to protect their citizens. This abhorrent behavior was answered at the WCTOH by the establishment of a legal defense fund courtesy of Michael Bloomberg and Bill Gates. Clearly, there is bold new thinking going on among some governments and public-spirited deep-pocketed entities [9].

The supply side of the tobacco epidemic has until recently been almost ignored in tobacco control; MPOWER and the FCTC mainly address the demand side of tobacco control (however, smuggling and illegal trade are now part of a new FCTC protocol). Trade agreements are one of the more insidious avenues that may sustain the global supply side of tobacco use, and the Trans-Pacific Partnership (TPP) is a case in point. This massive trade agreement is now being negotiated by the United States, China, and other partners around the Pacific Rim but, until recently, with little input on public health concerns for liberalized tobacco trade [10]. Following the lead of Malaysia, several negotiating parties now support the exclusion of tobacco trade from the agreement in order to assure the acceptability of public health interventions against tobacco use by participating nations [11]. Opening unrestricted trade throughout the Pacific region to tobacco products would defeat decades of progress made by Asian countries and greatly benefit the multinational tobacco industry. However, tobacco is not like any other commercial product. When used as directed, it kills half its users, and moreover, it creates extraordinary health and social costs that will not be made up by profits generated by open trade in tobacco products under the TPP. Tobacco should be excluded from free trade agreements so that participating countries can enact health policies that protect their citizens from tobacco-related diseases.

Environmental concerns are another growing focus in global tobacco control. At the WCTOH, considerable attention focused on the plight of poor tobacco farmers around the globe, in particular on how they are subject to human rights abuses, pesticide exposures, and unfair commercial arrangements with buyers. In addition, renewed attention was raised on the environmental impacts of tobacco growing, with pesticide use, land degradation, and deforestation cited as concerns [12]. New evidence has also been developed around the issue of “thirdhand smoke” (THS) exposure [13], on the impact of tobacco product waste on the environment [14], and on the possibility of developing an extended producer responsibility (EPR) framework to internalize the environmental costs of tobacco production and use back to the tobacco industry [15]. EPR includes financial, physical, and informative responsibilities and, ultimately, liability for all environmental damages caused by tobacco production and use.

Turning the responsibility for the environmental consequences of tobacco away from communities and individuals and back onto the tobacco industry would further address the supply side of the tobacco epidemic. The entire life cycle of tobacco manufacture and use should be seen as a longitudinal environmental hazard that adversely impacts the natural environment, the lives of tobacco growers, the condition of human habitations and communities, and ultimately human health.

Finally, substantial progress is being made in tobacco regulatory policy and science. In the US, the Family Smoking Prevention and Tobacco Control Act was signed into law in 2009, at long last providing the US Food and Drug Administration (FDA) with authority to regulate tobacco products, their labeling, and the market entry of new or altered tobacco products [16]. This legislation calls for using scientific evidence in the regulation of tobacco products in order to prevent false and misleading advertising and labeling, the entry of risky new products into the US market, and the use of inappropriate claims for harm reduction capabilities of new products. The FDA has partnered with the National Institutes of Health to fund an extensive network of 14 Tobacco Centers of Regulatory Science (TCORS) that will conduct important scientific research on regulatory-related issues and train a new generation of tobacco-control scientists [17]. This scientific endeavor promises to be as interesting as the regulatory process itself.

Certainly, substantial progress has been made in tobacco control since the publication of the 1962 Royal College of Surgeons Report in the UK and the 1965 Report of the US Surgeon General on the Health Consequences of Smoking. However, tobacco-related diseases are a global pandemic, increasingly affecting poor countries where unfinished agendas in infectious diseases continue to wreak havoc. The United Nation’s 2015 Millennium Development Goals did not address tobacco use or the growing epidemics of noncommunicable diseases [18]. Therefore, new global efforts are now needed in the post-2015 global health agenda to reduce the burden of these diseases, starting with those caused by tobacco. Tobacco control and the FCTC must be integrated into the proposed UN Sustainable Development Goals (SDGs), thereby mobilizing governments, scientists, and citizens to target the end of the tobacco epidemic. Cross-disciplinary work involving infectious diseases (HIV, tuberculosis [TB], and lower respiratory tract infections), maternal and child health, and environmental exposures must be linked to tobacco control efforts to ensure future progress in these areas as well. The endgame against tobacco will indeed demand something new, bold, and completely different from what has been done until now, and thus nothing should be off the table post 2015. With this in mind, let the endgames begin!

## Abbreviations:

EPR: extended producer responsibility
FCTC: Framework Convention on Tobacco Control
FDA: Food and Drug Administration
SDGs: Sustainable Development Goals
SHS: secondhand smoke
TB: tuberculosis
TCORS: Tobacco Centers of Regulatory Science
THS: thirdhand smoke
TPP: Trans-Pacific Partnership
WCTOH: World Conference on Tobacco or Health
WHO: World Health Organization
